# Editorial

**Published:** 1860-04

**Authors:** 


### EDITORIAL.

### FIRST ANNUAL COMMENCEMENT OF MEDICAL DEPARTMENT OF LIND UNIVERSITY.

According to previous notice, the Public Commencement of this new Medical Institution, was held on the evening of March 5th, 1860, in the Second Presbyterian Church.

At the hour appointed, the Church was well filled with citizens, embracing many professional men, and friends of education generally. The exercises commenced with prayer, by the Rev. Dr. Patterson, after which the candidates for graduation were called upon the platform, when Mr. J. S. Jewell, one of their number, delivered a very appropriate and interesting farewell address in behalf of the students. The President of the Faculty then announced the award of a premium to Rufus D. Cogswell, of Illinois, for the best Inaugural Thesis, and another to A. D. Andrews, of Wisconsin, for the second best Thesis. Prof. H. A. Johnson, on behalf of the Board of Trustees, conferred the Degree of Doctor of Medicine on the following gentlemen:

C. DeHaven Jones, of Evanston, Illinois.
John Conant, of Rockford, Illinois.
J. S. Jewell, of Attilla, Illinois.
Rufus D. Cogswell, of Wilmington, Illinois.
Lucien Ashley, of Magnolia, Illinois.
Thos. J. Rigg, of Chicago, Illinois.
J. F. Hopkins, of Milwaukee, Wisconsin.
A. D. Andrews, of River Falls, Wisconsin.
J. M. Kendall, of Wabash, Indiana.

The *ad eundum* degree was conferred on Drs. Edward C. Dickinson and Ezra A. Steele, both of Chicago, Illinois. After the conferring of the Degrees, Titus Deville, M. D., Professor of Anatomy in the University, announced his intention of returning to Europe, and delivered to the Faculty, Graduates and Students, a very appropriate and eloquent farewell address.

The regular Valedictory Address to the Graduates, was given by Prof. H. A. Johnson, on the qualifications of the true physician. The theme was presented in its most comprehensive aspect; embracing the physical, intellectual, and moral qualities necessary for the attainment of a just eminence in the medical profession. The several topics were admirably arranged; the illustrations and comparisons were chosen with that nice discrimination for which the speaker is justly celebrated; and the whole was clothed in language as pure and classic as it was eloquent and stirring. It was received by the audience and class with marked attention and pleasure.

The exercises in the Church were closed with a benediction: and the Trustees, Faculty, Graduates, Students, and invited guests, retired to the residence of Prof. N. S. Davis, where they partook of an entertainment in honor of the graduates, and spent two or three hours in the most pleasant social and intellectual enjoyment.

As an evidence of the latter (the intellectual) part of the entertainment, we append the sentiments offered, and the names of the respondents to each.

By Dr. J. F. Hopkins: "The *Lind University*, with a foundation as broad as the liberality of its *chief. almoner*, and as stable as the pillars of virtue; her Medical Department is plainly destined to be the great centre of medical science and instruction in the North-West." Responded to by S. Lind, B. W. Raymond, and Rev. Mr. Spencer.

By Dr. A. D. Andrews: "The *Faculty* of the Medical Department of the Lind University, neither corroded by the rust of old fogyism, nor soured by "green-eyed jealousy," nor blinded by an over-zealous spirit of reform, but genuine advocates of *rational improvements*, may their success be equal to their talents and their virtues." Responded to by Professors Byford, Hollister, and Taylor.

By Dr. C. D. Jones: "The *Senior* Professor of the *Senior* Department, Prof. Davis—the founder of the American Medical Association, and the personification of indomitable energy—may the throbings of his generous heart meet a cordial response from the profession at large, and he live to realize his highest ambition." Responded to by Prof. Davis; who in concluding his remarks, gave in return a sentiment to the *Senior Class*, which called out a very appropriate reply in their behalf from Dr. Jones.

By Dr. J. M. Kendall: "The *Senior* Professor of the Junior Department, Prof. H. A. Johnson—self-made and self-reliant; an example worthy of imitation. May the sentiments he has uttered find a lodgement in prolific minds, that will prove lasting monuments to perpetuate his name." Responded to by Prof. Johnson, who in return gave a sentiment to the *Junior Class;* which called out a very humorous and pleasant reply from Mr. Webber of that department.

By Dr. J. F. Hopkins: "Prof. E. Andrews, the accomplished and energetic Surgeon of the North-West." Responded to by Prof. Andrews.

By Dr. R. D. Cogswell: "Our *emeritus* Professor of Obstetrics, Dr. David Rutter, honored and revered by all who know him; may we never forget the good council he has given us."

By Dr. Hopkins: "Our esteemed *Hostess*, Mrs. Dr. Davis, one of the *true-hearted women of America.* We shall ever think of her as our memories linger around this festive scene. May she long live an ornament to society." Responded to by Prof. Spafford.

Throughout the evening everything was done in admirable order, and it was altogether the most interesting and pleasant college commencement we ever attended.

Immediately after the close of the regular annual college term, the Medical Faculty of the University arranged a course of summer instruction for those students who should remain in the city. A good text-book in each department of medical science was selected, and the student required to undergo a regular examination in each, accompanied by a familiar explanatory lecture once a week. By this arrangement the student gets one thorough examination in some branch every day. The dissecting room has also been kept open and supplied with *material*, under the charge of the Demonstrator. In addition to this course of systematic reading, examinations and dissections, they have one hour of clinical instruction every day by Professors Andrews and Davis, viz: On Mondays, Tuesdays, Thursdays and Fridays, in the Mercy Hospital, and on Wednesday and Saturday in the City Dispensary, at the University rooms.

Thus, the division of the regular College term into Junior and Senior Departments, adapted to the systematic and progressive advancement of the students; the lengthening of the term in both, to *five* months; the more comprehensive presentation cf the whole field of medical science and art by the devided term, and the increased number of Professors; and the continuance of the examinations, demonstrations, and clinical instruction, throughout the entire summer, from the close of one term to the commencement of the next; makes up a system of medical instruction for the Medical Department of Lind University, more comprehensive in its scope; more accurately adjusted in its details to the wants of the student in the successive steps of his pupilage; and more continuous, than is afforded by any other institution with which we are acquainted, in America.

The course of summer instruction is now in active progress, and regularly attended by a class of good students.

---

### ILLINOIS STATE MEDICAL SOCIETY.

We are assured by Dr. S. York, that the profession and citizens of Paris, have made ample arrangements to entertain hospitably all the members of the State Society who will attend the coming meeting on the second Tuesday in May next.

We hope there will be a full attendance from all parts of the State.

---

### DELEGATES TO THE STATE SOCIETY.

The Chicago Academy of Medical Sciences appointed the following delegates to the State Society, viz: S. C. Blake, Chas. G. Smith, Thomas Bevan, and J. H. Rauch.

The Chicago Medical Society, at its last annual meeting, appointed the following officers:

*President*—Orrin Smith, M. D.
*Vice President*—L. P. Cheeney, M. D.
*Secretary*—J. Swayne Wickersham, M. D.

